# Preoperative Assessment of Upper Extremity Secondary Lymphedema

**DOI:** 10.3390/cancers12010135

**Published:** 2020-01-06

**Authors:** Itay Wiser, Babak J. Mehrara, Michelle Coriddi, Elizabeth Kenworthy, Michele Cavalli, Elizabeth Encarnacion, Joseph H. Dayan

**Affiliations:** Division of Plastic and Reconstructive Surgery, Department of Surgery, Memorial Sloan Kettering Cancer Center, New York, NY 10065, USA; wiser125@gmail.com (I.W.); mehrarab@mskcc.org (B.J.M.); coriddim@mskcc.org (M.C.); eokenworthy@gmail.com (E.K.); cavallim@mskcc.org (M.C.); encarnae@mskcc.org (E.E.)

**Keywords:** lymphedema, preoperative assessment, indocyanine green, L-DEX, ULL-27, LLIS, patient reported outcomes, limb volumes, MRA

## Abstract

*Introduction:* The purpose of this study was to evaluate the most commonly used preoperative assessment tools for patients undergoing surgical treatment for secondary upper extremity lymphedema. *Methods:* This was a prospective cohort study performed at a tertiary cancer center specializing in the treatment of secondary lymphedema. Lymphedema evaluation included limb volume measurements, bio-impedance, indocyanine green lymphography, lymphoscintigraphy, magnetic resonance angiography, lymphedema life impact scale (LLIS) and upper limb lymphedema 27 (ULL-27) questionnaires. *Results:* 118 patients were evaluated. Limb circumference underestimated lymphedema compared to limb volume. Bioimpedance (L-Dex) scores highly correlated with limb volume excess (r^2^ = 0.714, *p* < 0.001). L-Dex scores were highly sensitive and had a high positive predictive value for diagnosing lymphedema in patients with a volume excess of 10% or more. ICG was highly sensitive in identifying lymphedema. Lymphoscintigraphy had an overall low sensitivity and specificity for the diagnosis of lymphedema. MRA was highly sensitive in diagnosing lymphedema and adipose hypertrophy as well as useful in identifying axillary vein obstruction and occult metastasis. Patients with minimal limb volume difference still demonstrated significantly impaired quality of life. *Conclusion:* Preoperative assessment of lymphedema is complex and requires multimodal assessment. MRA, L-Dex, ICG, and PROMs are all valuable components of preoperative assessment.

## 1. Introduction

Advances in microsurgical techniques and an improved understanding of the pathophysiology of lymphedema have led to the development of a variety of surgical options for the treatment of secondary lymphedema [1,2,3,4,5]. However, although numerous investigators have published outcomes following lymphovenous bypass [6,7,8,9], vascularized lymph node transplantation [2,3,10,11,12,13,14], and liposuction [5,15], a detailed discussion of the assessment of prospective surgical candidates has not been reported. As a result, there are currently many preoperative assessment protocols complicating comparison of outcomes between centers [16,17,18,19,20,21,22]. In addition, the rationale, utility, and comparative efficacy of preoperative tests for lymphedema has not been formally reviewed. This has resulted in duplication of effort, patient inconvenience, and increased resource costs. Finally, although it is generally agreed that patient reported outcomes are an important assessment tool for plastic surgery patients [23], the utility and baseline assessment of patients with secondary lymphedema of the upper extremity presenting for surgical evaluation has not been performed using validated questionnaires. 

Over the past 5 years, we have prospectively evaluated patients with secondary lymphedema of the upper extremity presenting for surgical consultation using a structured intake note, detailed physical examination, imaging techniques, and, more recently, 2 validated lymphedema questionnaires [24,25,26]. This experience has greatly increased our understanding of the pathological changes of lymphedema enabling us to streamline our approach and systematically record the preoperative information that has the highest yield for the treatment of this patient population. 

The purpose of this study was to critically review our preoperative assessment protocol for patients presenting for surgical evaluation of upper extremity secondary lymphedema and to provide a streamlined template for patient evaluation. In addition, we correlated preoperative measures of lymphedema and validated patients’ reported outcome questionnaires with lymphedema stage to understand the relative contribution of physical findings and patients’ reported symptoms to the pathophysiology of the disease. 

## 2. Materials and Methods

### 2.1. Study Design

This was a prospective cohort study conducted at the Memorial Sloan Kettering Cancer Center. We had approval from Memorial Sloan Kettering IRB #16–275, and the original approval date was 13 April 2016. Using our prospectively managed patient database, we identified all patients evaluated for surgical management of unilateral upper extremity swelling following axillary surgery between 1 January 2015 and 1 July 2018. Patient consent was not required; this was data pulled from our registry.

### 2.2. Limb Volume Measurement

A perometer (Pero-system model 1000NT, Wuppertal, Germany) was used for automated limb volume measurements. In order to minimize variability, perometer measurements were only performed by designated and trained clinical staff. The patient was positioned facing the perometer while placing the measured limb at 90-degree angle relative to the body and parallel to the machine. This position minimizes limb fluid shift that might skew the measurement. The proximal limb measurement point was taken at the distal border of the axilla to avoid volume distortion by including the deltoid muscle. The limb length on the affected side was matched to the contralateral limb. 

Limb volumes were also assessed using manual circumferential measurements and calculation of limb volumes using the truncated cone formula [27]. With the patient standing and upper limb held straight toward the floor, limb circumferences were measured at 4 cm intervals, from the wrist to 44 cm proximally. A truncated cone formula was then used to calculate limb volume: *V* = *h* (*C1*^2^ + *C1*⋅*C2* + *C2*^2^)/12π, where *V* is the volume of one 4 cm interval, C1 and C2 are the circumferences at either end of the interval, and h is the distance between them [27]. A difference of greater than 10% was considered diagnostic of lymphedema [28]. 

Finally, to calculate the sensitivity and specificity of limb circumference measurements without limb volume calculations, we recorded the circumference of the normal and affected limbs at points located 12 and 32 cm above the wrist. 

### 2.3. Bio-Impedance Measurement

Bio-Impedance measurements were obtained on all patients in the morning between 8–11 AM using the L-Dex model U400 (Impedimed, Brisbane, Australia). Bioimpedance spectroscopy is a technique which detects lymphedema by measuring the electrical impedance of the limb. Extracellular fluid content is extrapolated from this measurement (the higher the fluid content, the lower the impedance) and applied to a normative dataset from which an “L-Dex” score is derived. A score of greater than 10 is considered diagnostic for lymphedema [29,30]. 

### 2.4. Quality of Life Measurement

Assessment of health-related quality of life was performed using two validated patient reported outcome measurement (PROM) questionnaires. The Lymphedema Life Impact Scale (version 2) [24] includes 18 questions distributed across physical, functional and psychological domains. The upper limb lymphedema 27 (ULL-27) [26] is specific for the upper extremity and includes 27 questions distributed across physical, emotional, and social domains. Both LLIS and ULL-27 PROM are presented as impairment score (%), where a score of 0% means no impairment and 100% the most severe impairment. These scores are presented per specific domain and for overall impairment. We used two different PROMs because they have different lookback periods: The LLIS asks the patient about the past week, while the ULL-27 questions refer to symptoms over the past month.

### 2.5. MRA Imaging

The pre-operative work-up also included magnetic resonance angiography of the chest and upper extremities. This test was performed to assess the fluid and fat composition of the limb, evaluate for venous stenosis, and rule out occult malignancy. All MRAs were performed by the same radiologist and were obtained on a 1.5 Tesla MRI (GE Healthcare, Waukesha, WI, USA) using a dedicated peripheral vascular coil with the patients in the supine position. Coronal acquisition was obtained with T1-weighted post-contrast imaging without fat saturation using gadofosveset trisodium contrast agent. Axial and sagittal high-resolution T1 fat saturation gradient echo images of both upper extremities after contrast administration were obtained. Intravascular gadolinium agent was used, and delayed phase vascular imaging was obtained. 

### 2.6. Lymphoscintigraphy

Upper extremity lymphoscintigraphy was performed bilaterally by first injecting 0.5 mL of 1% plain lidocaine followed by 0.2 mL of filtered technetium 99 into the first and third webspaces. Images were acquired every 30 min up to 3 h post-injection. The presence of dermal reflux, or lack of radiotracer uptake in the axilla after 3 h, was considered abnormal and consistent with lymphedema.

### 2.7. ICG Lymphography

ICG lymphography was performed bilaterally with subdermal injection of 0.2 mL of 1% plain lidocaine followed by 0.1 mL of indocyanine green dye (2.5 mg/mL aqueous solution) into the first and third webspaces. Images were acquired 30 min following injection using near-infrared fluorescence lymphography (SPY-PHI, Stryker Corporation, Kalamazoo, MI, USA). Pathological changes noted on ICG lymphography were classified using the staging system previously reported by Yamamoto et al. [31]. Briefly, a linear pattern is normal, while splash, stardust, and diffuse patterns represent abnormalities of the lymphatic system with increasing levels of deterioration in lymphatic function. The presence of any of these abnormal patterns was considered to represent impaired lymphatic function.

### 2.8. Statistical Methods

Statistical analysis was conducted using SPSS software v.25 (SPSS technologies, IBM, Armonk, NY, USA). Continuous variables were presented as averages and standard deviations and were compared using independent student t-test. Categorical variables were presented as counts and percentage of total and were compared using Fisher exact test. Normal distribution was assessed using the Kolmagorov-Smirnov test. Multiple group comparison of continuous variables was performed using analysis of variance (ANOVA) followed by a multiple comparison analysis using LSD test. Linear correlation was assessed using the Spearman correlation test. ULL-27 scores were inverted to align with the LLIS score.

## 3. Results

### 3.1. Patient Demographics

A total of 118 patients with unilateral upper extremity secondary lymphedema were included in the study (Table 1). The average age was 54 ± 11 years, while the average BMI was 26.1 ± 3.9 kg/m^2^. Most patients (*n* =116; 98.3%) were women, and the most common cause of lymphedema was breast cancer treatment (*n* = 96, 81.3%). The average duration of lymphedema was 41 ± 54 months, and the average time to develop lymphedema following surgery was 23.8 ± 57.1 months. The vast majority of patients had International Society of Lymphology (ISL) stage 1 or 2. Briefly, ISL staging is as follows: stage 0: subclinical lymphedema, 1: mild lymphedema that improves with elevation, 2a: moderate pitting edema, 2b: non-pitting lymphedema, and 3: elephantiasis and irreversible skin changes.

The most common referral source was self-referral (35.6%), followed by breast surgeon (21.2%), other plastic surgeons (19.5%), oncologist (11.9%), and physical/occupational therapist (6.8%). The most common compression regimen frequency was around the clock compression (*n* = 55, 47%); 12 (10%) patients did not use any compression therapy. 

**Figure 1:**
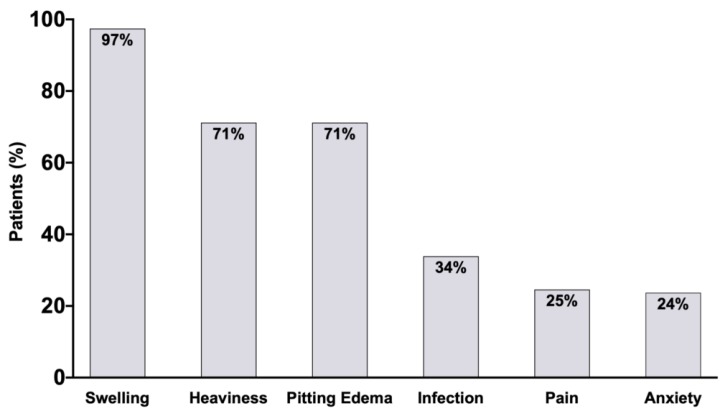
Extremity lymphedema presenting signs and symptoms.

The most common signs and symptoms of lymphedema at the time of evaluation in our series is presented in Figure 1. Swelling was reported by 115 patients (97.5%): Swelling of the hand by 68 patients (57.6%); forearm and elbow by 99 patients (83.9%); upper arm by 99 patients (83.9%). Fifty-six patients (47.5%) reported swelling of the entire upper extremity. Forty patients had a history of cellulitis of the affected extremity (33.9%); of these, 5 (4.2%) were on prophylactic antibiotics at the time of evaluation.

Other symptoms included heaviness (*n* = 84, 71%), pitting edema (*n*= 84, 71%), history of infections (*n* = 40, 33.9%), pain (*n* = 29, 25%), and anxiety (*n* = 28, 24%). Common findings on the clinical exam, other than swelling and pitting edema, included upper extremity sensory impairment (*n* = 19, 16%), motor impairment (*n* = 10, 9%), and shoulder joint limited range of motion (*n* = 45, 38%). 

### 3.2. Limb Volume Measurements Are More Sensitive and Specific than Circumferential Measurements

Because limb swelling was the most common manifestation of lymphedema, a considerable amount of time was spent evaluating the most common techniques being used to quantify this symptom. There is significant heterogeneity in the literature on this topic: Some groups measure limb volume using a perometer, some use manual circumferential measurements and derive limb volume using a truncated cone formula, and others quantify swelling using circumference measurements alone [28].

**Figure 2:**
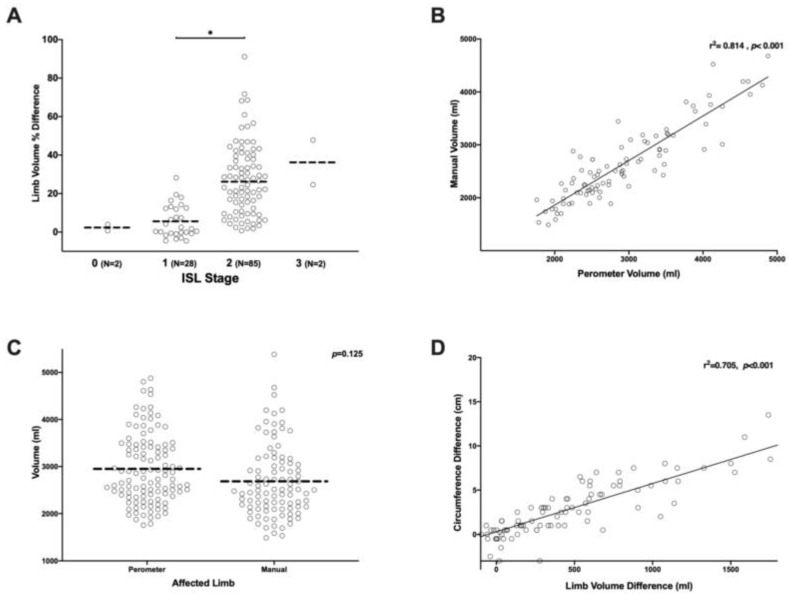
(**A**) Scatter plot of upper extremity limb volume difference (%) distribution by ISL stages. * *p* < 0.05. (**B**) Correlation plot of upper extremity volume measurement using manual vs. perometer techniques. (**C**) Scatter plot of upper extremity volume measurement using manual vs. perometer techniques. (**D**) Correlation plot of upper extremity volume vs. circumference measurement.

The average percent increase in limb volume in our patients based on ISL staging was as follows: Stage 0 = +2.3 ± 2.3%; stage 1 = +5.6 ± 8.5%; stage 2 = +26.2 ± 19.1%; stage 3 = +36.2 ± 16.4% (Figure 2A). The difference between stage 1 and 2 was statistically significant (*p* < 0.001). However, there were only 2 patients in this series with ISL stage 0 or stage 3 lymphedema. Limb volume and ISL stage were positively correlated, but this correlation was very weak (r^2^ = 0.235). Stated differently, limb volume increases were only responsible for approximately 23.5% of the variance in lymphedema stage. This data suggests that ISL staging is not granular enough to stratify patients with a diagnosis of lymphedema if limb volume is considered the most important presenting symptom. 

Consistent with previous studies, we found that perometer measurements were highly correlated with volume measures derived from circumferential measurements using the truncated cone formula (r^2^ = 0.814, *p* < 0.001; Figure 2B) [27]. However, manual measurements tended to underestimate the total volume as compared to the perometer (Figure 2C). The average limb volume assessed by perometer measurement was 2,466 ± 451 mL while the average for manual measurement was 2,216 ± 433 mL. Although this difference was not statistically significant, this finding suggests that measurements should be performed consistently using the same method over time and that volume measurements obtained with the perometer are not interchangeable with those derived from manual circumferential measurements. 

Perometer measurements of limb volume difference were also correlated with upper (32 cm above the wrist) circumferential measurements (r^2^ = 0.705, *p* < 0.001; Figure 2D). This finding is not surprising given the high degree of correlation between manual measurements and the perometer. However, it is likely that single measurements are more prone to error. To determine the sensitivity, specificity, and positive predictive value of single (12 cm proximal to the wrist) or dual-site (12 cm and 32 cm proximal to the wrist) circumferential measurements, the commonly used >2 cm difference was used as diagnostic of lymphedema. The >2 cm circumference difference was then compared to the commonly accepted diagnostic threshold for limb volume difference by >10% assessed by perometer. This analysis revealed that circumferential measurements had a relatively low sensitivity (82.8%) and specificity (85.3%) as compared with limb volume measurements. In fact, circumferential measurements had only a 74.4% positive predictive value, suggesting that using circumference measurements alone tends to under-diagnose and under-estimate the degree of lymphedema (Table 2)

### 3.3. Bio-Impedance Measurements Are a Sensitive Means of Diagnosing Early Stage Lymphedema

**Figure 3:**
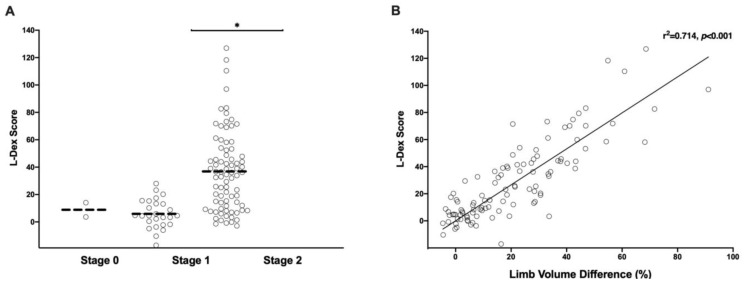
(**A**) Scatter plot of upper extremity L-Dex score distribution by ISL stages; * *p* < 0.05. (**B**) Correlation plot of limb volume difference (%) vs. L-Dex score.

Abnormal L-Dex scores defined as greater than 10 were recorded in 71 (65.7%) patients. The L-Dex score significantly correlated with both ISL stage (r^2^ = 0.521, *p* < 0.001, Figure 3A) and limb volume difference (%) (r^2^ = 0.714, *p* < 0.001, Figure 3B). Compared to limb circumference measurements, L-Dex was more sensitive in diagnosing lymphedema (91.2% vs. 83.5%) and had a higher positive predictive value (87.3% vs. 76.2%) when using a limb volume difference of greater than 10% as the diagnostic threshold. However, L-Dex scores were less specific (77.5% vs. 94.1%) than circumferential measurements (see Table 2). This finding is consistent with the concept that L-Dex measurements may be useful for early-stage diagnosis of lymphedema, but these measurements may also reflect non-specific swelling in some cases. Taken together, our findings with L-Dex measurements suggest that this is a highly sensitive test for preoperative assessment of patients evaluated for surgical treatment of lymphedema.

### 3.4. Even Minor Changes in Limb Volume can Have Significant Effects on PRO Measures

Forty-nine patients completed the LLIS, and of these, 42 patients also completed the ULL-27 questionnaire. In both tests, a score of 0 is equivalent to no impairment, while a score of 100 is consistent with complete impairment. The average percent impairment reported the LLIS was 40.7 ± 20.7% for the physical, 35.5 ± 22.1% for the psychological, and 34.8 ± 19.5% for the functional scales. The overall impairment score for the LLIS was 37.2 ± 18.8%. Percent impairment scores for the ULL-27 were 39 ± 24% for the physical domain, 40 ± 23% for the psychological domain, and 26 ± 22% for the social domain (Table 3). The overall impairment score for the ULL-27 was 36.8 ± 20.3%.

**Figure 4:**
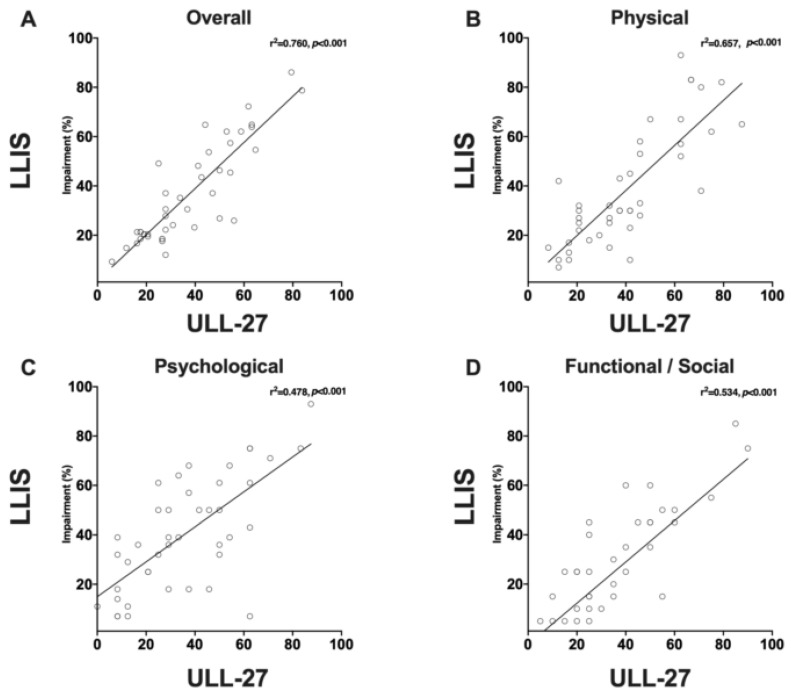
(**A**) Correlation plot of ULL-27 vs. LLIS total impairment score (%). (**B**) Correlation plot of ULL-27 vs. LLIS physical domain impairment score (%). (**C**) Correlation plot of ULL-27 vs. LLIS psychological domain impairment score (%). (**D**) Correlation plot of ULL-27 social vs. LLIS functional domain impairment score (%).

The correlation between the individual domains (Physical: r^2^ = 0.657, *p* < 0.001; Social/functional: r^2^= 0.534, *p* < 0.001; Psychological; r^2^ = 0.478, *p* < 0.001; Figure 4A–C) was also statistically significant, though these correlations were weaker than the overall impairment. There was a significant correlation between overall impairment as measured by the LLIS and the ULL-27 (r^2^ = 0.76, *p* < 0.001; Figure 4D)**.** Taken together, these results suggest that patients with upper extremity lymphedema have significant impairment in all domains tested by both tests.

**Figure 5:**
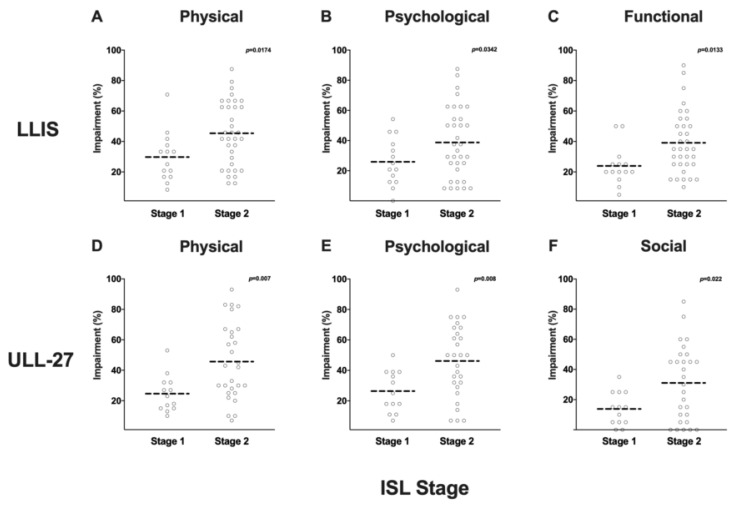
(**A**) Scatter plot of ISL stage vs. LLIS physical domain impairment score (%). (**B**) Scatter plot of ISL stage vs. LLIS psychological domain impairment score (%). (**C**) Scatter plot of ISL stage vs. LLIS functional domain impairment score (%). (**D**) Scatter plot of ISL stage vs. ULL-27 physical domain impairment score (%). (**E**) Scatter plot of ISL stage vs. ULL-27 psychological domain impairment score (%). (**F**) Scatter plot of ISL stage vs. ULL-27 social domain impairment score (%).

We next analyzed the correlation between LLIS and ULL27 scores and ISL stage (Figure 5A–F). Patients with ISL stage 2 lymphedema had significantly higher disability scores in all domains as compared with individuals with stage I disease (physical: 45 ± 21% vs. 30 ± 16%, *p* = 0.01; psychological: 39 ± 23% vs. 26 ± 16%, *p* = 0.03); functional: 39 ± 20% vs. 24 ± 13%, *p* = 0.01). We noted a similar pattern in the ULL27 instrument (physical: 46 ± 25% vs. 25 ± 12%, *p* = 0.006; psychological: 46 ± 24% vs. 26 ± 14%, *p* = 0.008); social: 31 ± 25% vs. 14 ± 11%, *p* = 0.02).

**Figure 6:**
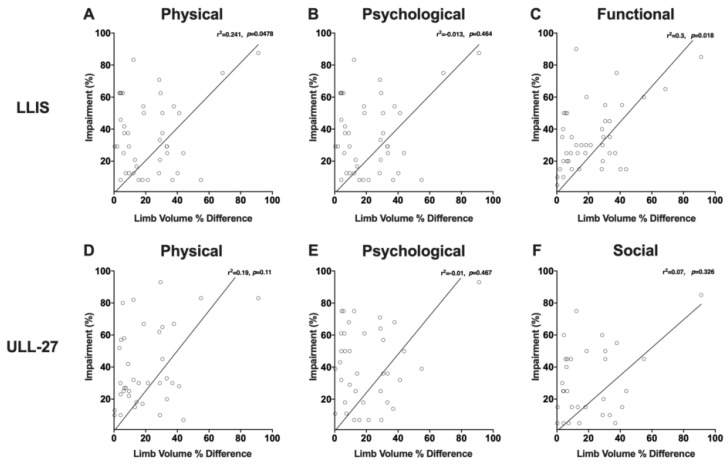
(**A**) Correlation plot of limb volume difference (%) vs. LLIS physical domain impairment score (%). (**B**) Correlation plot of limb volume difference (%) vs. LLIS psychological domain impairment score (%). (**C**) Correlation plot of limb volume difference (%) vs. LLIS functional domain impairment score (%). (**D**) Correlation plot of limb volume difference (%) vs. ULL-27 physical domain impairment score (%). (**E**) Correlation plot of limb volume difference (%) vs. ULL-27 psychological domain impairment score (%). (**F**) Correlation plot of limb volume difference (%) vs. ULL-27 social domain impairment score (%).

We next sought to determine if PRO measures were correlated with changes in limb volume excess (Figure 6). LLIS physical and function scores were significantly but weakly correlated with limb volume excess (r^2^ = 0.24, *p* = 0.048, Figure 6A and r^2^ = 0.3, *p* = 0.018, Figure 6C respectively). Stated differently, limb volume changes in our patients were only responsible for approximately 20–30% of the variance in physical and functional disability. In contrast, the correlation between limb volume difference (%) and psychological impairment as measured by the LLIS was not statistically significant (Figure 6B). In fact, some of our patients displayed significant psychological (as well as physical and functional) impairment, even with modest increases in limb volume. The ULL27 domains had an overall similar slope and pattern of distribution, however, the correlation between limb volume excess and reported measures in physical, psychological, and social domains were not statistically significant (Figure 6D–F).

Taken together, our findings with PROM in patients with secondary lymphedema of the upper extremity suggest that the LLIS may be more sensitive than the ULL27 for measuring the degree of physical and functional disability resulting from lymphedema limb volume excess. Our findings also show that the correlation between physical/functional impairment and increases in limb volume excess is weak suggesting that even minor increases in limb volume can have a significant impact on the quality of life measures of some patients with upper extremity lymphedema. This hypothesis is supported by the fact that we found no correlation between psychological impairment and increased limb volume difference. These findings suggest that analysis of PRO is an important aspect of preoperative and postoperative measures in patients who are slated to undergo lymphatic surgery. 

### 3.5. Lymphoscintigraphy Has a Low Specificity and Positive Predictive Value for Lymphedema Diagnosis but May Be Useful for Surgical Planning

Seventy-nine patients (67%) underwent lymphoscintigraphy as part of their initial evaluation in our center (Table 4). Axillary lymph node uptake was absent in the affected limb in 53 (67%) patients, and delayed uptake was documented in 26 (33%) patients. Dermal backflow was present in 24 (30%) patients.

The validity measures of any pathological findings in lymphoscintigraphy (i.e., lack of axillary uptake or the presence of dermal backflow) are presented in Table 2. The sensitivity of lymphoscintigraphy to diagnose lymphedema in patients with a minimum limb volume excess of 10% was 88% and the positive predictive value was 72.1%. However, the specificity and negative predictive value of lymphoscintigraphy were low (41.4% and 66.7%, respectively). These results suggest that lymphoscintigraphy alone is insufficient in some cases to diagnose lymphedema. However, this test may have utility in identifying patients who have axillary lymph node uptake thus guiding the degree of scar excision and axillary dissection that may be performed safely in the course of a vascularized lymph node transplant. Thus, in patients with axillary lymph node uptake is demonstrated by lymphoscintigraphy, a more prudent axillary scar excision may be necessary to prevent injury to the remaining functional lymphatic vascular network. 

### 3.6. ICG Lymphography Is Highly Sensitive for Detecting Lymphedema

Ninety- seven patients (82%) underwent outpatient ICG lymphography as part of their lymphedema initial evaluation (Table 4). Every patient in this study had a presence of a pathologic ICG pattern in the limb with lymphedema. Stage 1 (Splash pattern) was observed in 20 patients (20.6%), stage 2 (Stardust pattern) in 62 patients (63.9%) and stage 3 (diffuse) in 15 patients (15.5%). In contrast, all normal limbs in this study had a normal linear ICG pattern. 

**Figure 7:**
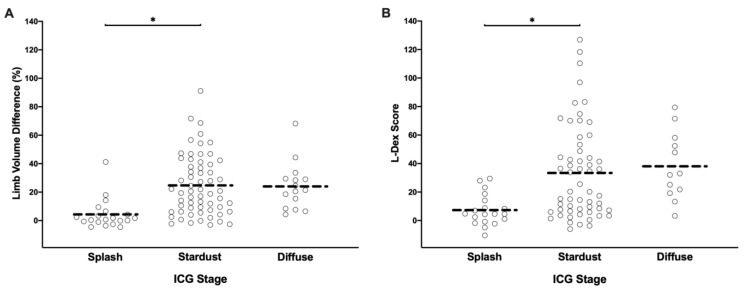
(**A**) Scatter plot of limb volume difference (%) distribution by ICG stages. (**B**) Scatter plot of L-Dex score distribution by ICG stages. * *p* < 0.05.

Both L-Dex score and limb volume did not significantly correlate with ICG stage. Patients with the early stage splash patterns on ICG tended to have lower L-Dex scores and lower limb volumes, but aside from this trend, there was no tight correlation (Figure 7A,B). This supported the authors’ observation that limb volume and related extracellular fluid content do not necessarily reflect the physiology of the abnormal lymphatic system. Interestingly, there were patients with fairly advanced abnormal ICG patterns that had a minimal limb volume difference of less than 10% and an L-Dex score of less than 10. ICG may uncover deeper pathology than appreciated if only assessing volume and bioimpedance, but this is a subject requiring further study.

**Figure 8:**
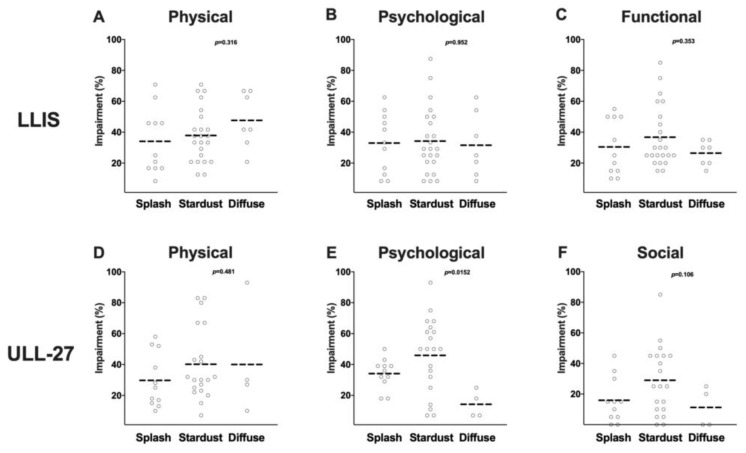
(**A**) Scatter plot of ICG stage vs. LLIS physical domain impairment score (%). (**B**) Scatter plot of ICG stage vs. LLIS psychological domain impairment score (%). (**C**) Scatter plot of ICG stage vs. LLIS functional domain impairment score (%). (**D**) Scatter plot of ICG stage vs. ULL-27 physical domain impairment score (%). (**E**) Scatter plot of ICG stage vs. ULL-27 psychological domain impairment score (%). (**F**) Scatter plot of ICG stage vs. ULL-27 social domain impairment score (%).

There was no correlation between ICG staging and changes in patient reported outcomes in either the LLIS or the ULL27 (Figure 8A–F). The average impairment score for all three domains was not significantly different in patients with splash, stardust, or diffuse patterns suggesting that ICG staging is not predictive of the degree of disability resulting from lymphedema. This may be due to the limitations of the current ICG staging system, which is entirely subjective and not quantitative. The current system only provides a one-time snapshot of the limb. It does not quantify clearance or lymphatic transport which would be a more meaningful measure of lymphatic function. ICG is an effective means for directly visualizing the superficial lymphatic collectors and is an area requiring further investigation.

### 3.7. MRA Imaging Is Useful for Identification of Abnormalities in Venous Outflow and Diagnosis of Lymphedema 

Table 4 summarizes the findings of MRA performed during the initial evaluation. Seventy-eight patients underwent upper extremity MRA (66.1%) and of these, 12 (15.4%) had evidence of narrowing or stenosis in the axillary vein. One patient (0.8%) had a partial thrombus in her axillary vein. Identifying venous pathology is important because it may contribute to limb swelling and may also compromise the results of lymph node transplant or lymphovenous bypass if not corrected. 

Evidence of fluid accumulation was found in 64 patients (82%), and fat hypertrophy was found in 60 patients (77%). One patient in this series was found to have occult bony metastasis on MRA. Of note, an additional patient was found to have incidental bony metastasis on CT angiogram but was not included in these results because she did not have an MRA due to a tissue expander in place. Evidence of fluid accumulation or fat hypertrophy on MRA was highly sensitive for the diagnosis of lymphedema as defined by a limb volume excess of ≥10% (94.2% and 96.2% sensitive, respectively). In other words, nearly every patient who had an increase of 10% or more in their limb volume demonstrated fluid accumulation or fat hypertrophy on MRA. However, these findings had a lower specificity for lymphedema (44% and 64%, respectively). This finding suggests that the pathological changes in lymphedema are progressive and that arbitrary cut-offs in changes in limb volume are not reflective of this process. MRA evidence of fluid accumulation also had relatively high negative and positive predictive values (78.6% and 77.8%, respectively). The negative and positive predictive values were even higher in patients with evidence of fat hypertrophy on MRA (84.8% and 88.9%, respectively) suggesting that MRA is also useful for confirming the diagnosis of lymphedema. 

## 4. Discussion

### 4.1. ISL Staging Is Subjective and Not Useful for Preoperative Classification 

ISL staging has long been used to classify the severity of lymphedema. However, several recent studies have addressed the lack of correlation of ISL stage to measures of lymphedema such as limb volume difference and L-Dex score [32,33]. This study demonstrated that while ISL stage loosely correlated with various lymphedema measures, including limb volume difference, L-Dex scores, PROM, and ICG stage, these correlations were weak. This likely reflects inherent limitations of staging a disease based solely on physical exam findings, particularly if the disease has a progressive and proliferative nature. Clinical staging for lymphedema remains highly subjective and coarse, limiting its usefulness to only describing extremes along a broad spectrum of disease. This limits its use in stratifying patients and tracking meaningful changes following surgical or medical interventions and underscores the need for a more sophisticated staging system in the future. 

### 4.2. Limb Volume Measurements Are More Sensitive than Circumferential Changes

Various clinical standards have been described to diagnose lymphedema, including an increase of 2 or more centimeters in circumference measurements or an increase in limb volume of greater than 10%. However, a variety of diagnostic thresholds have been reported and used in the literature [28]. Nevertheless, limb volume increase is an important pathologic feature of lymphedema, and its reduction is a main goal of surgical management of lymphedema for most patients. 

Based on the results in this study, limb volume measurements are clearly preferable to circumference measurements as the latter underestimates the degree of lymphedema. The same technique, whether it be perometer or truncated cone formula, should be used longitudinally when assessing changes in limb volume over time. 

### 4.3. Bio-Impedance Is Sensitive and Specific but Requires Optimization for Reproducible Results

The bio-impedance or L-Dex score is reflective of fluid accumulation in the limb and is a sensitive tool for early detection of lymphedema. [29,34,35,36] Consistent with previous reports, our analysis showed L-Dex was significantly correlated with ISL stage and limb volume excess [30,37]. L-Dex measurements also had a high degree of sensitivity and a high positive predictive value for the diagnosis of lymphedema. However, in contrast to prior studies demonstrating a low level of inter and intra-observer variability, we found that L-Dex measurements required a significant amount of training and optimization for consistent results. Changes in ambient temperature, humidity, sweating, patient preparation, and activity had significant results on measurements, and a standardized approach was required for reproducible results. In addition, consistent with previous studies, we found that L-Dex measurements were highly influenced by compression garment use [37]. Thus, although L-Dex scores are reliable and correlated with the severity of limb volume excess, this analysis is easily influenced by external factors and should be optimized and inter/intra user variability should be measured. However, it should be noted that the U400 device was used to assess bioimpedance in this study which requires adhesive pads and leads, allowing for greater variability. These variability issues have largely been addressed by the newer Sozo device which will be adopted for future study. In summary, the L-Dex score was found to be the most rapid and reliable non-invasive method for detecting early-stage lymphedema in this study.

### 4.4. PROMs

PROMs are an important tool for analyzing outcomes for a variety of reconstructive procedures. However, the use of validated measures for assessment of lymphedema symptomatology has not been widely adopted. Several validated measures are available for the assessment of patient reported outcomes in lymphedema. Some tools, such as the Disabilities of Arm Shoulder and Hand questionnaire (DASH), are non-specific and measure the degree of disability resulting from functional changes in the upper extremity resulting from lymphedema [38,39]. Other outcome measures, such as the LLIS, lymphedema quality of life questionnaire (LYMQOL), or the ULL27, have been specifically developed to assess the degree of disability in various scales resulting from lymphedema [24,25,40].

Previous studies that compared lymphedema PRO questionnaires to objective measurements found variability in their association with objective lymphedema measures. Lee et al. [33] compared quality of life scores of 54 lower and upper extremity lymphedema patients using DASH, LEFS and LYMQOL questionnaires with their ISL stage and L-Dex score. Their study found no correlation between PROM and objective measurements. These results were consistent with a previous study by Tsauo, who compared the international classification model for disability and health scores with clinical data of 61 breast cancer related lymphedema patients. Tsau found that patient’s symptoms rather than limb volume difference are significantly correlated with upper arm function, again demonstrating the limited ability of limb volume difference as a clinical tool to assess lymphedema severity [41].

The results in this study did demonstrate some modest correlation between LLIS and ULL27 with limb volume excess and lymphedema stage. However, limb volume was not the most dominant factor in predicting quality of life. There were a significant number of patients in this study with only minimal limb volume difference and early lymphedema stage who reported a high degree of impairment in quality of life. This effect was particularly striking in physical/functional and psychological domains and suggests that lymphedema symptoms are a major cause of morbidity for patients. These findings suggest that limb volume, bioimpedance, and ISL stage are inadequate in assessing the lymphedema patient, and PROMs is an important tool for providing a more complete picture. In addition, although the disability scores recorded by the LLIS and ULL27 were highly correlated, the findings in this study suggest that the LLIS is more sensitive for measuring the degree of physical and functional disability.

### 4.5. MRA Imaging

MRA was found to be a very useful tool on multiple levels as it is highly sensitive in detecting lymphedema by identifying fluid in the subcutaneous tissues which is not normally present. It also directly reveals the degree of lymphedema-related fat hypertrophy which is a significant confounding variable when treating these patients [42,43]. MRA revealed 15% of patients in this study had venous stenosis. This finding in our practice is further assessed with either duplex ultrasound or venography. Venous pathology is important to identify because venous hypertension may not only contribute to limb swelling but also compromise the effects of lymphovenous bypass or lymph node transplant [44]. Finally, occult metastatic disease was also picked up on MRA. The findings in this study confirm the value of MR, particularly when embarking on lymphatic surgery. 

### 4.6. Lymphoscintigraphy

Lymphoscintigraphy is considered by many clinicians the gold standard in diagnosing lymphedema [45]. Although several studies found the sensitivity and specificity of lymphoscintigraphy to be as high as 95% and 100% respectively [46], others had conflicting evidence. For example, Maclellan et al. [45] found that dermal backflow appeared only in 36% of patients diagnosed with lymphedema. They also did not find any correlation between delayed tracer transit to the regional nodes and limb volume excess. The results in this study demonstrated a similar incidence in dermal backflow in only a minority of patients. Absence of lymph node uptake alone may not be sufficient as we have observed patients without any clinical lymphedema to have absent uptake in the regional node basin making this finding questionably useful. The findings in this study and the work of Maclellan, et al. highlight the limitations of lymphoscintigraphy in the diagnosis of lymphedema. Nonetheless, we have found lymphoscintigraphy to be useful in the preoperative assessment because if there are any functional nodes present, it is critical to avoid violating them particularly during lymph node transplant. Lymphoscintigraphy also provides a preoperative baseline to determine if the transplanted lymph nodes have uptake postoperatively.

### 4.7. ICG Lymphography

ICG lymphography has become the most widely used imaging modality among microsurgeons for lymphovenous bypass. It allows for preoperative staging and direct visualization of the lymphatic channels targeted for bypass. A number of staging systems have been proposed based on recurrent patterns of backflow. Yamamoto et al. have based an ICG staging system on dermal backflow patterns and their locations in the upper extremity [47]. Their study showed a significant correlation to clinical stage and lymphedema duration. Similarly, Chang et al. had proposed a staging system based on the number of lymphatic vessels visible and the presence of dermal backflow [48,49,50,51].

ICG lymphography appears to be the most sensitive test for lymphedema in this study. All abnormal limbs with a limb volume of >10% had abnormal ICG patterns. However, the specificity of this test has yet to be determined since this analysis would require evaluation of patients who underwent axillary lymph node dissection but did not develop clinical evidence of lymphedema. Furthermore, a quantitative ICG assessment, as opposed to subjective pattern-based staging system, will be essential for a more meaningful method of determining the degree of lymphatic impairment—a currently elusive grail in lymphedema.

### 4.8. Limitations

There are several limitations to this study. First, this patient cohort is composed of patients who, in most cases, had an established diagnosis of lymphedema. As a result, our findings cannot be compared with healthy controls or patients who had lymph node dissection and did not develop lymphedema. Although we tried to mitigate this problem by evaluating changes in the normal contralateral limb, future analysis of patients who underwent ALND but did not develop lymphedema is necessary to fully understand the specificity and sensitivity of the tests we used. Our cohort includes mainly lymphedema patients with ISL stage 1 and 2, and patients with either ISL stages of 0 or 3 are underrepresented. This limits the study interpretation and generalizability of our results. Future studies are planned and will address this issue by enrolling additional patients with early and late-stage disease.

## 5. Conclusions

Early identification and comprehensive evaluation are key for improved outcomes using conservative therapy and surgery. Preoperative evaluation of lymphedema requires a multi-modal approach as there is not a single metric that adequately represents the state of a patient’s disease. When lymphatic surgery is planned, assessment of not only the lymphatic system but also the venous system and fluid/fat composition are required to guide the most appropriate treatment. Given the results in this study, all of the modalities discussed have a role: (1) MRA for evaluation of the venous system, fluid/fat composition, and evaluation of occult recurrence; (2) ICG for screening potential candidates for LVA if they have early disease; (3) lymphoscintigraphy for evaluating preoperative and postoperative lymph node activity; (4) limb volume, (5) bioimpedance; and (6) PROMs. Prospective outcomes following surgery using these outcome measures is currently in progress and will further guide our understanding of the optimum recipe for preoperative evaluation. 

## Figures and Tables

**Table 1:** Baseline characteristics of patients presenting with upper extremity secondary lymphedema.

Characteristic		Average ± SD or No. ()
N		118
Right Upper Extremity		61 (51.7)
Age (Years)		54 ± 11
Female Sex		116 (98.3)
BMI		26.1 ± 3.9
Cancer Site	Breast	96 (81.3)
	Other	22 (18.7)
Lymphedema Duration (Months)		41 ± 54
Chemotherapy		112 (95)
Radiotherapy	Axilla	69 (59)
	Chest	101 (85.6)
Axillary Lymph Node Dissection	Complete	101 (85.6)
	Sentinel	14 (12)
	None	3 (2.4)
ISL Stage	0	2 (1.7)
	1	28 (23.8)
	2	85 (72)
	3	3 (2.5)
Referral Source	Self	42 (35.6)
	Breast Surgeon	25 (21.2)
	Plastic Surgeon	23 (19.5)
	Other	28 (23.7)
Compression Therapy	Around the Clock	55 (47)
	Day or Night	26 (22.2)
	Occasionally	24 (20.5)
	Never	12 (10.3)

**Table 2:** Specificity and sensitivity of assessments for diagnosis of lymphedema (volume difference >10%).

Test	Sensitivity	Specificity	Negative	Positive
Circumference Diff. >2 cm	82.8	85.3	90.6	74.4
L-Dex >10	91.2	77.5	83.8	87.3
Pathological changes on LS	88.0	41.4	66.7	72.1
MRA Fluid Accumulation	94.2	44.0	78.6	77.8
MRA Fat Hypertrophy	96.2	64.0	84.8	88.9

**Table 3:** Average impairment scores (%) of lymphedema life impact scale (LLIS) and upper limb lymphedema (ULL-27) patient reported outcomes.

Test	Physical	Domains Psychological	Functional (LLIS)/Social (ULL-27)	Overall
LLIS	40.7 ± 20.7%	35.5 ± 22.2%	34.8 ± 19.5%	37.2 ± 18.8%
ULL-27	38.8 ± 23.6%	40.4 ± 22.7%	25.6 ± 22.5%	36.8 ± 20.3%

**Table 4:** Imaging findings in upper extremity lymphedema.

Imaging Modality	Characteristic	No. (%)
Lymphoscintigraphy	Axillary Uptake at 3 h	26 (33)
	Dermal Backflow	24 (30.4)
ICG Lymphography	Stage 0 (“Linear”)	0 (0)
	Stage 1 (“Splash”)	20 (20.6)
	Stage 2 (“Stardust”)	62 (64)
	Stage 3 (“Diffuse”)	15 (15.4)
MRA	Fluid Accumulation	64 (82)
	Fat Accumulation	60 (77)
Recurrence		1 (1.3)
Axillary Vascular Abnormality		12 (15.4)
